# Development of a portable high-T_c_ spherical neutron polarimetry device at the Oak Ridge National Laboratory

**DOI:** 10.1038/s41598-025-02279-2

**Published:** 2025-05-17

**Authors:** Nicolas Silva, Tianhao Wang, Sichao Fu, Masaaki Matsuda, Barry Winn, Elynn An, James Beare, Lowell Crow, Kiman Park, Chenyang Jiang

**Affiliations:** 1 Neutron Technologies Division, Oak Ridge National Laboratory, Oak Ridge, TN 37830 USA; 2 Neutron Scattering Division, Oak Ridge National Laboratory, Oak Ridge, TN 37830 USA; 3 Present Address: National High Magnetic Field Laboratory, Tallahassee, FL 32310 USA; 4 Present Address: Spallation Neutron Source Science Center, Dongguan, 523803 Guangdong China

**Keywords:** Polarized neutron scattering, Spherical neutron polarimetry, Larmor precession, Adiabatic transition, Meissner effect, Characterization and analytical techniques, Characterization and analytical techniques

## Abstract

Spherical neutron polarimetry is a powerful polarized neutron scattering technique used to determine complex magnetic structures which are only partly accessible by other methods. This technique measures the full neutron polarization change upon scattering from a sample by fully decoupling the incoming and outgoing neutron polarization with a zero-field chamber placed at the sample position. Recent advancements and testing are presented for a new spherical neutron polarimetry device utilizing high-T_c_ superconducting YBCO films, PHiTPAD, at the High Flux Isotope Reactor at Oak Ridge National Laboratory. Furthermore, we introduce a conceptual design that utilizes wavelength-independent adiabatic transitions to adapt spherical neutron polarimetry for use with pulsed neutron sources, thereby expanding its potential applications in neutron scattering research.

## Introduction

Neutron polarization analysis is a widely used polarized neutron scattering technique to study magnetic structures and dynamics in materials in modern condensed matter physics and materials science. The Blume-Maleyev equations, which are the master equations that describe the theory of polarized neutron scattering from crystals, were developed in the 1960s^1,2^. Classical longitudinal polarization analysis (LPA), first demonstrated by Moon et al. in 1969^3^, and its derivative XYZ polarization analysis^4^ are well established methods in polarized neutron scattering that allow for the clear separation of nuclear, magnetic, and spin-incoherent scattering contributions. LPA also enables detailed magnetic structure analysis, allowing determination of precise spin orientations. For neutron spectroscopy, LPA is a powerful technique to separate magnon and phonon scatterings and separate longitudinal and transverse magnon modes, which is particularly important for studies of magnetic excitations in quantum and metallic materials^5–10^. Despite LPA’s great success in distinguishing between different types of scattering, it inherently misses transverse directional changes in neutron polarization, offering a limited view of the scattering event.

Spherical Neutron Polarimetry (SNP), introduced by the Cryogenic Polarization Analysis Device (Cryopad) at Institut Laue–Langevin (ILL) approximately 30 years ago, overcomes the limitations in LPA by exploiting the vectorial nature of neutron polarization^11,12^. Cryopad’s zero-field region, delineated by superconducting niobium films (T_c_ ~ 9 K), decouples incoming and outgoing neutron polarizations, enabling precise control over neutron polarization through adiabatic and non-adiabatic transitions. In contrast, LPA is constrained to evaluate scattered polarization states which are either parallel or antiparallel to an ambient magnetic field. Since its introduction, Cryopad has been upgraded several times and has been routinely utilized at several major neutron scattering facilities in Europe and Japan. Following Cryopad, the MuPAD device, developed with a mu-metal zero-field chamber for room-temperature polarization control, was built in the 2000s for use at the Paul Scherrer Institute in Switzerland and the FRM-II in Germany^13,14^.

Recently at Indiana University in the United States, a prototype miniature SNP device called CryoCUP was developed by taking advantage of high-T_c_ superconducting yttrium-barium-copper-oxide (YBCO) films as Meissner screens^15^. When the YBCO film is in the Meissner state, the magnetic-flux density **B** at the very surface is forced to run parallel (tangential) to that surface, making YBCO an effective magnetic shield. Compared to niobium, YBCO has a much higher critical temperature T_c_ of around 93 K, which renders cooling of such films much easier and simpler than that of niobium films in Cryopad. In CryoCUP, a closed cycled refrigerator (CCR) is employed to quickly cool the YBCO films below their superconducting transition temperature T_c_, while in Cryopad liquid helium is used to cool the niobium films. CryoCUP has proved the feasibility of using YBCO films in a SNP device, however, it has several limitations: First, CryoCUP has very limited angle coverage and is only suitable for small angle scattering experiments; second, CryoCUP does not have an independent sample environment, i.e., the cryostat that cools the YBCO films also cools the sample which greatly limits the sample temperature range to between 20 K and 80 K. This limited temperature range puts a restriction on the number of samples that can be studied by CryoCUP.

To overcome these limitations in CryoCUP, a new compact wide-angle spherical neutron polarimetry device PHiTPAD (Portable High-Tc Polarization Analysis Device has been developed at the Oak Ridge National Laboratory (ORNL). Compared to CryoCUP, PHitPAD accesses a much wider range of scattering angles (5° to –80°), enabling comprehensive coverage for a vast array of magnetic neutron scattering studies. It features a mu-metal zero-field chamber to minimize magnetic interference (< 2 mG residual field at the sample position) as well as an independent liquid helium cryostat for broader sample temperature control. Designed for adaptability across various neutron instruments at the High Flux Isotope Reactor (HFIR), PHiTPAD’s compactness enhances its portability. Notably, PHiTPAD is the first full-fledged SNP device in North America and is now available in the user program of HFIR at the polarized triple axis spectrometer (PTAX). This paper details PHiTPAD’s conceptualization, construction, and operational testing.

SNP has been routinely used at reactor-based neutron sources, but not yet at pulsed neutron sources. The devices currently in use for SNP are specifically engineered for monochromatic neutron beams and narrow solid angle acceptance for the scattered neutrons, rendering them incompatible with the broad spectrum and pulsed nature of neutron beams at spallation sources and the larger solid angle acceptance of their detector arrays. To bridge this gap, a redesign of SNP devices is required, involving significant modifications or entirely new designs, to make them adaptable to the unique conditions of pulsed neutron beamlines. In this paper, we introduce a conceptual design of a SNP device tailored for use on the Hybrid Spectrometer (HYSPEC) at the Spallation Neutron Source (SNS).

## Design and construction of PHiTPAD

PHiTPAD is mainly designed for PTAX^16^, which is equipped with Heusler crystals that function as the neutron polarizer and the polarization analyzer^17^. The design of PHiTPAD considered space constraints at PTAX as well as future portability across multiple instruments at HFIR.

**Fig. 1:**
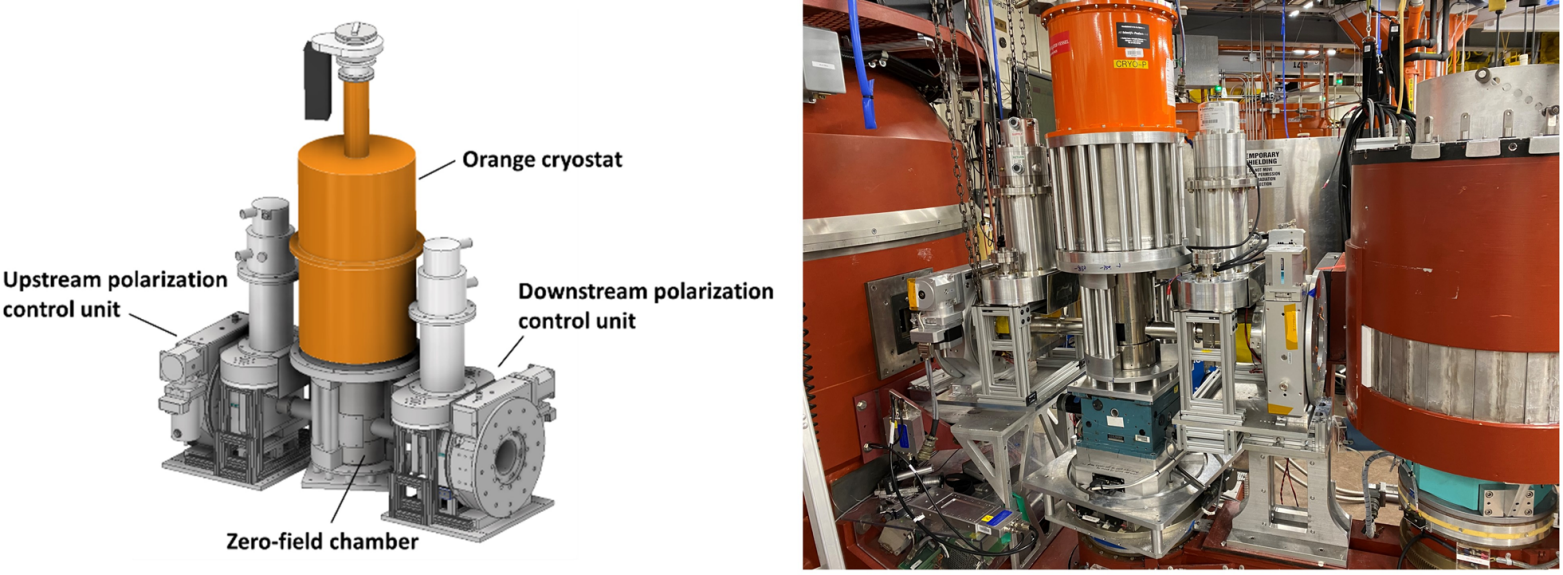
Left, the schematics of PHiTPAD; right, PHiTPAD set up on the PTAX beamline at HFIR.

**Fig. 2:**
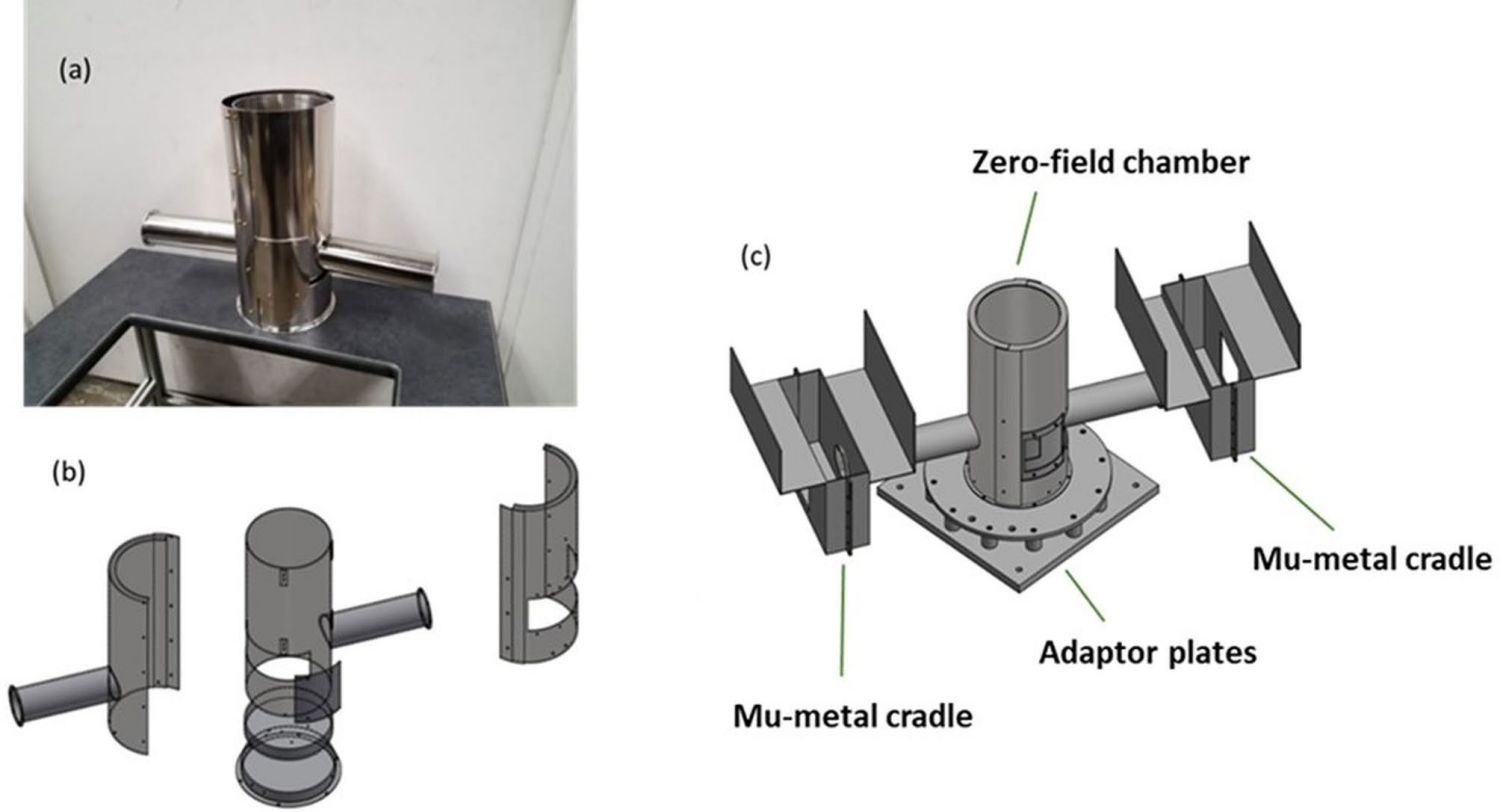
(**a**) A photograph of the zero-field chamber by itself. (**b**) An exploded rendering of the zero-field chamber, where the outer subassembly is at left, right and bottom, with an optional mu-metal patch. The inner subassembly is shown intact in the middle-top. (**c**) A rendering of the zero-field chamber attached to the mu-metal cradles for the incident beam and scattered beam precession regions, and the adaptor plates which both enable mounting the SNP device on PTAX’s sample goniometer and include attachment points which support the flange-mountable orange cryostat which controls sample temperature.

PHiTPAD comprises three major parts: a zero-field chamber, two polarization control units (one for upstream and one for downstream neutrons), and an orange cryostat, as shown in Figure 1. The overall length of PHitPAD is 105 cm. The zero-field chamber (Fig. 2a, b) enables independent control of both incoming and outgoing neutron polarizations. It utilizes a dual-layer concentric mu-metal design, enhancing magnetic field isolation. This chamber features an outer mu-metal subassembly anchored with respect to the incident beam, while the inner subassembly follows the scattered beam arm, accommodating a wide angular range of +5° to −80°. Each subassembly has a vertical cylindrical mu-metal component coinciding with sample axis, and a horizontal mu-metal cylinder which surrounds either the incident (outer chamber) or scattered (inner chamber) beam and connects to the mu-metal cradles for the incident beam and scattered beam precession regions (Fig. 2c). Both subassemblies are open to the cryostat at 210 mm above the sample position, and the outer subassembly is closed at the bottom with mu-metal. The horizontal slot on the outer cylinder permits free movement of the inner chamber’s scattered beam cylinder and can be patched with additional small mu-metal pieces to close the gap. The measured residual field at the sample position inside the zero-field chamber is lower than 2 mG, and the estimated field integral is ~ 0.1 Gauss cm, corresponding to a polarization rotation of 0.265° per angstrom.

**Fig. 3:**
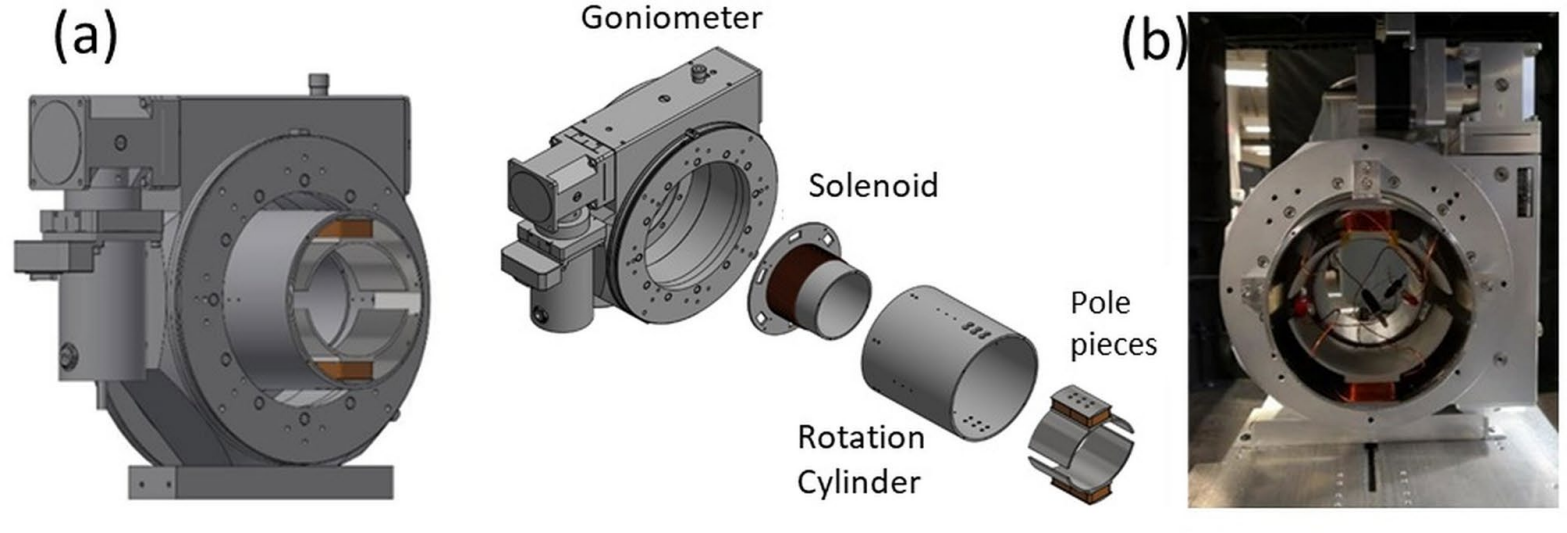
(**a**) The schematic and exploded view of the nutator. (**b**) The assembled nutator.

Two degrees of freedom are needed to orient neutron polarization into any direction in space. In MuPAD, the polarization control is realized by two orthogonal, tunable room-temperature magnetic precession coils^13,14^. It is also possible to use magnetic thin films like Permalloy films as precession regions^18–20^. PHiTPAD realizes neutron polarization control by combining adiabatic and non-adiabatic transitions of the polarization in magnetic fields. Each polarization control unit is composed of a nutator and a precession region. The nutator rotates the neutron polarization adiabatically into a plane perpendicular to the neutron flight direction, and the polarization turns non-adiabatically (Larmor precession) in the precession region. Figure 3 shows the schematic and assembly of the nutator. The whole nutator unit can be broken down into several components: a horizontal axis solenoid guide field, a rotatable magnetic field region, and a Huber^®^ 420 goniometer. The solenoid guide field generates a magnetic field in the longitudinal direction along the neutron beam path which can be coupled to other magnetic fields on the beamline. The rotatable magnetic field region consists of a rotation cylinder, two soft iron pole pieces each connected to a rectangular coil, and a mu-metal ring providing shielding and field return. Each pole piece is shaped like a half circle, and the two pole pieces are arranged to generate a uniform field in the transverse direction of the neutron beam. The guide field, pole pieces and mu-metal ring are all connected to a rotation cylinder that is mounted to the non-magnetic Huber^®^ 420 goniometer. This mechanism enables a full 360° rotation with high precision and repeatability. The current in the solenoid guide field and the pole piece coils can be adjusted to achieve an optimal adiabatic condition for different neutron wavelengths, so that polarized neutrons can preserve orientation (parallel or antiparallel) with respect to a spatially gradually changing guide field direction. The nutators are placed right next to the outside faces of the cradles shown in Fig. 2b.

**Fig. 4:**
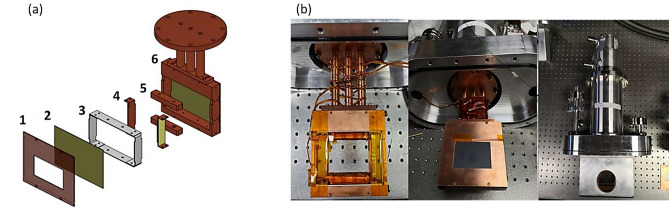
(**a**) Exploded rendering of the precession region, with 1. Front copper cover plate, 2. One of two YBCO films on sapphire substrate, 3. Mu-metal frame, 4. Side YBCO strips mounted on copper pieces, 5. Copper blocks for the YBCO conducting tape windings, and 6. Copper cyogenic attachment to the CCR with additional pieces like 1 & 2 attached to the back. (**b**) Left shows components 3-6 but without the second YBCO film, middle shows the full assembly illustrated in (**a**) but with the top flange of the vacuum chamber also shown, and right shows the fully enclosed vacuum chamber with CCR cold head; the windows utilitzed on the outer vacuum chamber are sapphire windows.

Each precession region that sits inside one of the mu-metal cradles contains two high-T_c_ YBCO films, a pair of superconducting coils and a copper frame as shown in Fig. 4a. Each YBCO film is coated on a 100 × 90 × 0.5 mm sapphire substrate and is also covered with a 100 nm gold protection layer. When cooled below its critical temperature of 90 K, the YBCO film serves as a Meissner shield that defines a sharp magnetic boundary for the precession region. The copper frame has a rectangular opening for the YBCO film. A cryogenic mu-metal frame is embedded inside the copper frame and two additional YBCO strips are attached to the copper framing pieces on the side. The superconducting coils are wound around the copper piece attached to the mu-metal frame on the top and bottom, providing a well-defined vertical field in the middle. All these components are housed in an aluminum vacuum chamber and are cooled by a cryogen-free Sumitomo CH-110 single stage closed-cycle refrigerator (CCR) (Fig. 4b). The cooling system of PHiTPAD is greatly simplified compared to that in Cryopad. With an air-cooled compressor, the base temperature in the precession region can go down to ~25 K, far below YBOC’s critical temperature.

The custom top-loading orange cryostat from A.S. Scientific Products Ltd. provides a versatile sample environment for PHiTPAD with a temperature range of 1.5–300 K. Liquid nitrogen and liquid helium are consumed in the cryostat to cool the sample. The orange cryostat has a narrow tail (OD 80 mm) at beam elevation with a 50 mm ID sample well in it. A fiberglass sample stick ensures a completely non-magnetic environment at the sample position. The stick at the upper vacuum interface is mounted on a vertical axis Aerotech^®^ rotation stage so the sample orientation angle can be adjusted freely. At the bottom of the sample stick is mounted a cryogenic Attocube^®^ nano-goniometer, which is a piezo-based tilting stage to allow for sample tilting with a tilting range up to 5° in two orthogonal directions. The sample stick is also configured such that voltage/current can be applied to the sample if necessary. The whole orange cryostat is supported by an aluminum frame as shown in the setup on PTAX in Figure 1.

## Calibration and operation

PHiTPAD was first assembled offline in the lab to check the performance of each component. The zero-field chamber maintained a near zero-field (< 2 mG) at any angle within its rotation range. The rotatable magnetic field portion of the nutator rotates freely with high precision driven by a Galil^®^ motion controller, which takes about 30 s to complete a 360° rotation. Each precession region achieved vacuum via a turbo pump, and the precession region cooled to the base temperature of ~25 K in 4 h. The coils in each precession region were powered by a Lakeshore 625 bipolar superconducting magnet power supply, and the current in the precession coils was tested up to 20 A without quenching. Bipolar Kepco^®^ 20-20D power supplies were used to power the solenoid guide and rotatable magnetic field coils of the nutators.

Online calibration with neutrons is crucial to ensure the performance and validity of the device. It is necessary to do a calibration every time PHiTPAD is reinstalled on a neutron instrument. The calibration begins after the mechanical integrity is checked and the precession regions are zero-field cooled. The direct beam is used for calibration due to its high flux. Our calibration procedure is similar to that used for Cryopad^21,22^. The calibration procedure involves three steps: alignment between the upstream and downstream nutators, calibration of the upstream and downstream precession regions, and alignment between the nutators and the precession regions.

**Fig. 5:**
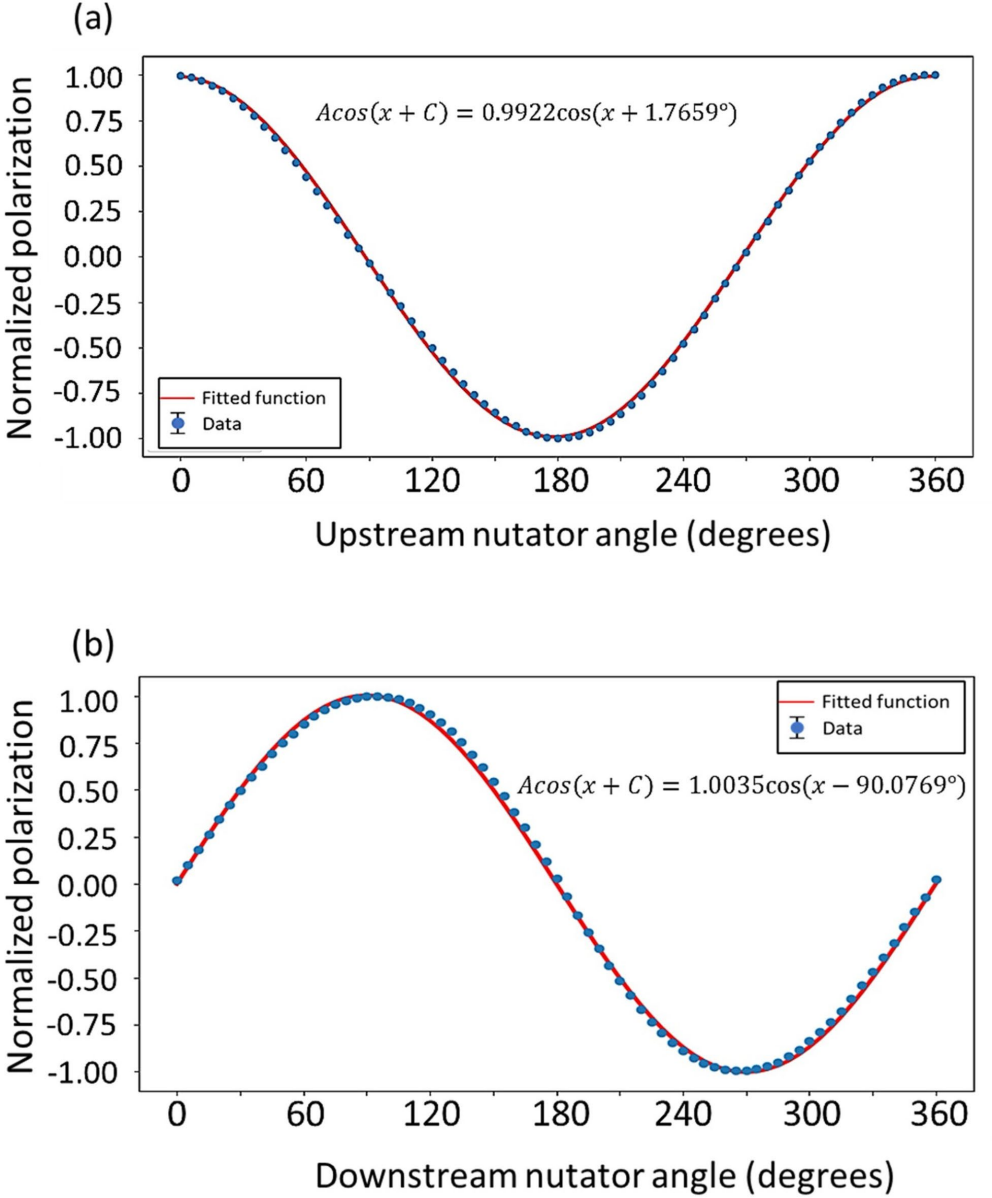
(**a**) The intial 360° scan of the upstream nutator with downstream nutator fixed at 0° postion. (**b**) The 360° scan of the upstream nutator with downstream nutator fixed at 90° postion after the initial misalignment adjustment. The error bars in the figures are smaller than the data point markers.

The nutators’ alignment is performed first. Current is turned off in both precession regions, and the downstream nutator is fixed at the nominal 0° position such that its field is in the vertical direction. The neutron polarization is then measured as a function of the upstream nutator rotation angle from 0 to 360°. The measured polarization is fit to a cosine function with a period of 360°, and the phase in the fitting indicates the relative misalignment angle between the two nutators. Figure 5a shows an alignment measurement for the nutators with a fitted misalignment angle of 1.77°. The goniometer on the nutator has an accuracy better than 0.01°. Thus, it is easy to align the two nutators by resetting the zero positions once the misalignment angle is known. After the initial adjustment, another scan can be done to test the alignment by rotating the downstream nutator to 90° and then repeating the 360° scan of the upstream nutator. Figure 5b presents a measurement following the initial alignment adjustment using the angle derived in Fig. 5a. The fitted phase of 90.08° indicates good perpendicularity between the two nutators.

**Fig. 6:**
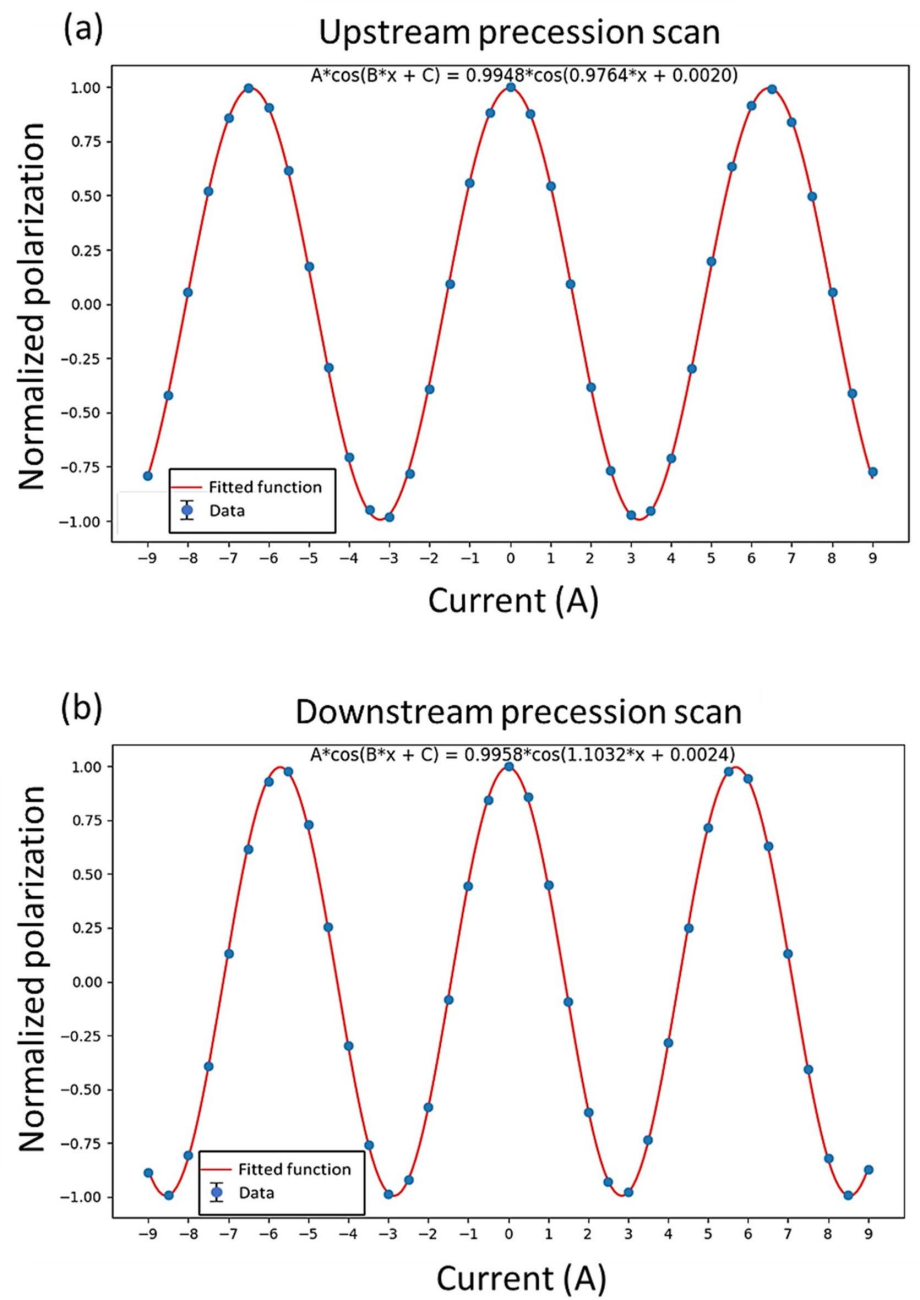
(**a**) Upstream precession scan. (**b**) Downstream precession scan. The error bars in the figures are smaller than the data point markers.

The second step is to calibrate the two precession regions. To do this, the two nutators are rotated to the 90° position so that the nutator fields are perpendicular to the precession fields. The aim is to measure the neutron polarization as a function of the injected current in the precession regions. The upstream precession region is usually calibrated first with the downstream precession coil turned off. By varying the input current in the upstream precession coil over a large range with a fixed interval, a polarization vs current plot is measured and fit to a cosine function, from which the precession constant can be determined in the unit of radians per ampere (Fig. 6a). This constant can also be normalized to the neutron wavelength used at the instrument, facilitating the computation of the precession angle for any neutron wavelength. The calibration of the downstream precession region follows the same procedure with the upstream precession coil turned off (Fig. 6b).

**Fig. 7:**
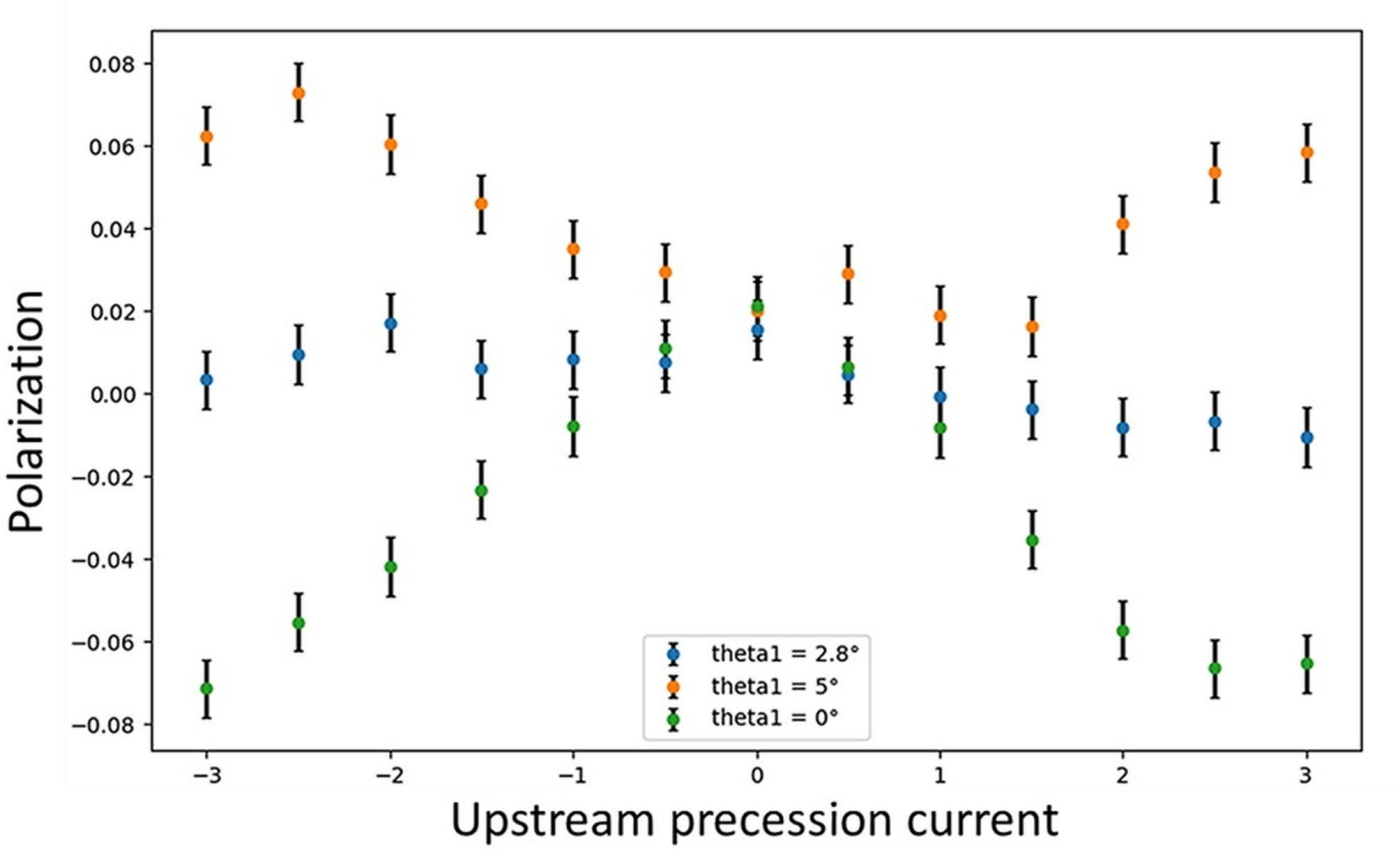
Upstream precession current scans aiming to align the upstream nutator and upstream precession region. The two nutator fields are positioned perpendicular to each other in each scan and the upstream nutator field is approximately in the vertical direction.

The last step aligns the nutators with respect to the precession fields. Since the precession regions cannot be readily rotated after installation, nutator angles are calibrated instead. To increase sensitivity, this calibration process ensures that the two nutator fields remain orthogonal to each other at all times during this step. For the upstream precession region, this means if the upstream precession field is aligned parallel with the upstream nutator, the measured polarization would be zero, and the polarization should be independent of the current in the upstream precession coil. Figure 7 shows three scans of the upstream precession current with the upstream nutator set at different angles, and the nutator at 2.8° yields the best outcome. Typically, a series of scans across a range of nutator angles are conducted and the method of least squares is employed to find the optimal angle. In principle, the same procedure should be applied to the downstream precession region. However, it is shown in the second step that the precession scans from both regions produced almost identical maximum polarizations and matching phases (Fig. 6), which is a direct proof of parallelism of the two precession fields. Consequently, calibrating the downstream region using the nutators is often skipped. Nevertheless, in the case that the parallelism of the two precession fields is found insufficient, shims can be inserted between the precession region and its support frame to make minor adjustments to the tilt angle.

No separate standalone spin flipper is used with PHiTPAD on the beamline PTAX due to space constraints. Instead, each polarization control unit in PHiTPAD can function as a cryo-flipper because the Meissner effect of the YBCO films used in the precession regions creates well-defined magnetic boundaries. For instance, to obtain a component of neutron polarization, measurements are initially conducted with the downstream nutator field oriented in one direction, followed by a subsequent measurement in the reverse direction. The nutator field direction can be easily switched by our bipolar power supply. This method effectively utilizes the existing structure for polarization control without the need for additional space-consuming equipment. The flipping efficiency of each control unit was measured to be better than 99.5% with the well-established two-flipper method, which is on par with other widely used high-efficiency neutron spin flippers.

## Test on PTAX

Following the setup and calibration of PHiTPAD on PTAX, a series of measurements of polarization matrix was conducted on various single-crystal samples, including silicon (Si), bismuth ferrite (BiFeO_3_) and Ca_2_Y_2_Cu_5_O_10_. All samples were first aligned using the neutron alignment station at HFIR before they were installed on the sample stick. On PTAX 13.5 meV neutrons were utilized for polarization measurements. The conventional polarization coordinate was used with *z* perpendicular to the scattering plane, *x* along the direction of the scattering vector, and *y* completing the right-handed coordinate system. To measure a polarization matrix for a given Bragg peak, the incident neutron polarization ***P***_*i*_ is oriented along the *x, y* and* z* directions respectively, and for each incident polarization the scattered polarization ***P***_*f*_ is measured along x, y and z directions. Therefore, a polarization matrix contains a total of 9 components, and its component *P*_*ij*_ (*i, j* = x, y, z) indicates the incident polarization is along the direction *i* and the measured polarization along the direction *j*.

Si is a strong nuclear scatterer and its (1 1 1) nuclear Bragg peak is accessible with PHiTPAD, which makes it a good candidate for a baseline test as coherent nuclear scattering does not change neutron polarization. BiFeO_3_, on the other hand, is a well-studied multiferroic material, which shows an antiferromagnetic ordering with a cycloidal order below its Néel temperature T_N_ ~ 640 K^23,24^. The magnetic peak (1/2 1/2 1/2) was investigated in our measurement. Since BiFeO_3_ has three equivalent magnetic domains, the measured polarization matrix would be domain averaged. Additionally, the test included the quasi-one-dimensional magnet Ca_2_Y_2_Cu_5_O_10_, which has an orthorhombic crystal structure. This material exhibits a long-range antiferromagnetic ordering below its Néel temperature T_N_ ~ 29.5 K, with magnetic moments along the *b*-axis^25^. The sample was oriented to measure the magnetic Bragg peak (0 0 1). We also included a polar and chiral sample NiCo_2_TeO_6_ from a recent user experiment with T_N_ ~ 52 K. In the antiferromagnetic phase NiCo_2_TeO_6_ has an incommensurate helical structure with spins lying in the ab-plane^26^. The magnetic peak (0 0 1.26) was measured. Table 1 shows the measured results of these samples and corresponding theoretical polarization matrices, and the measured polarization matrices agree well with the theoretical ones.

**Table 1:** Measured polarization matrices for silicon, BiFeO_3_, Ca_2_Y_2_Cu_5_O_10_ and NiCo_2_TeO_6 _in comparison with theoretical matrices.

Sample		Normalized polarization matrix			Theoretical polarization matrix		
	***P***_*f*_ ***P***_*i*_	x	y	z	x	y	z
Silicon	x	0.999 (3)	0.039 (4)	−0.025 (4)	1	0	0
	y	0.022 (4)	0.999 (3)	−0.014 (4)	0	1	0
	z	0.054 (4)	0.025 (4)	0.966 (3)	0	0	1
BiFeO_3_	x	−0.939 (7)	−0.017 (12)	0.006 (13)	−1	0	0
	y	0.029 (12)	0.036 (12)	0.025 (13)	0	0	0
	z	0.026 (12)	0.042 (12)	−0.014 (12)	0	0	0
Ca_2_Y_2_Cu_5_O_10_	x	−1.005 (6)	−0.003 (7)	−0.007 (7)	−1	0	0
	y	0.020 (7)	0.995 (6)	0.032 (7)	0	1	0
	z	0.002 (7)	−0.024 (7)	−0.962 (6)	0	0	−1
NiCo_2_TeO_6_	x	−0.993 (5)	0.117 (8)	0.013 (8)	−1	0	0
	y	−0.869 (7)	0.120 (10)	0.013 (11)	−1	0	0
	z	−0.863 (7)	0.111 (10)	0.008 (10)	−1	0	0

The test experiments conducted on PTAX have conclusively validated the efficacy of PHiTPAD, leading to its integration into HFIR’s user program since late 2022. During the test, several issues were also identified. First, malfunctions with the Attocube^®^ goniometer prevented its use in adjusting the sample’s tilt angle, potentially affecting the sample’s positioning. Furthermore, the operation of a high-field superconducting magnet at the adjacent VERITAS instrument caused interference with polarization measurements, attributed to the substantial stray magnetic fields. Additionally, occasional inconsistencies were observed with the Kepco^®^ bipolar power supplies, which struggled to achieve the predetermined set points. We are addressing these issues to improve the overall performance and reliability of PHiTPAD.

## SNP beyond HFIR

To date, existing SNP devices are designed for monochromatic neutron beams and are ill-suited for the broad neutron spectrum characteristic of a pulsed beam. A research team at ILL has proposed a design that utilizes double precession regions in conjunction with field ramping to align all polarization vectors for different neutron wavelengths to one direction^27^. To our knowledge, this method has yet to be realized in practice.

In this work we propose a novel conceptual design for a SNP device on the Hybrid Spectrometer (HYSPEC) at SNS^28,29^. HYSPEC is a direct-geometry spectrometer with a large detector bank of 60° horizontal angle coverage. It is equipped with a vertically focusing Heusler crystal array to monochromate and polarize the incident neutrons, and a supermirror array to analyze the neutron polarization. Because the incident beam of HYSPEC is still monochromatic, the new SNP design can reuse that of the upstream part of PHiTPAD, i.e., a combination of a nutator and a precession region.

However, a critical challenge arises with the scattered beam, which typically comprises neutrons of various wavelengths spreading across a broad horizontal angle. The rotation angle of polarization in a precession region is wavelength dependent. As a result, the old design would not work as no single static precession field could uniformly orient all polarizations of different neutron wavelengths to one direction. Conversely, the adiabatic transition of neutron polarization is generally wavelength independent, provided the adiabatic condition is satisfied. Our proposed design for the scattered beam capitalizes on this principle.

**Fig. 8:**
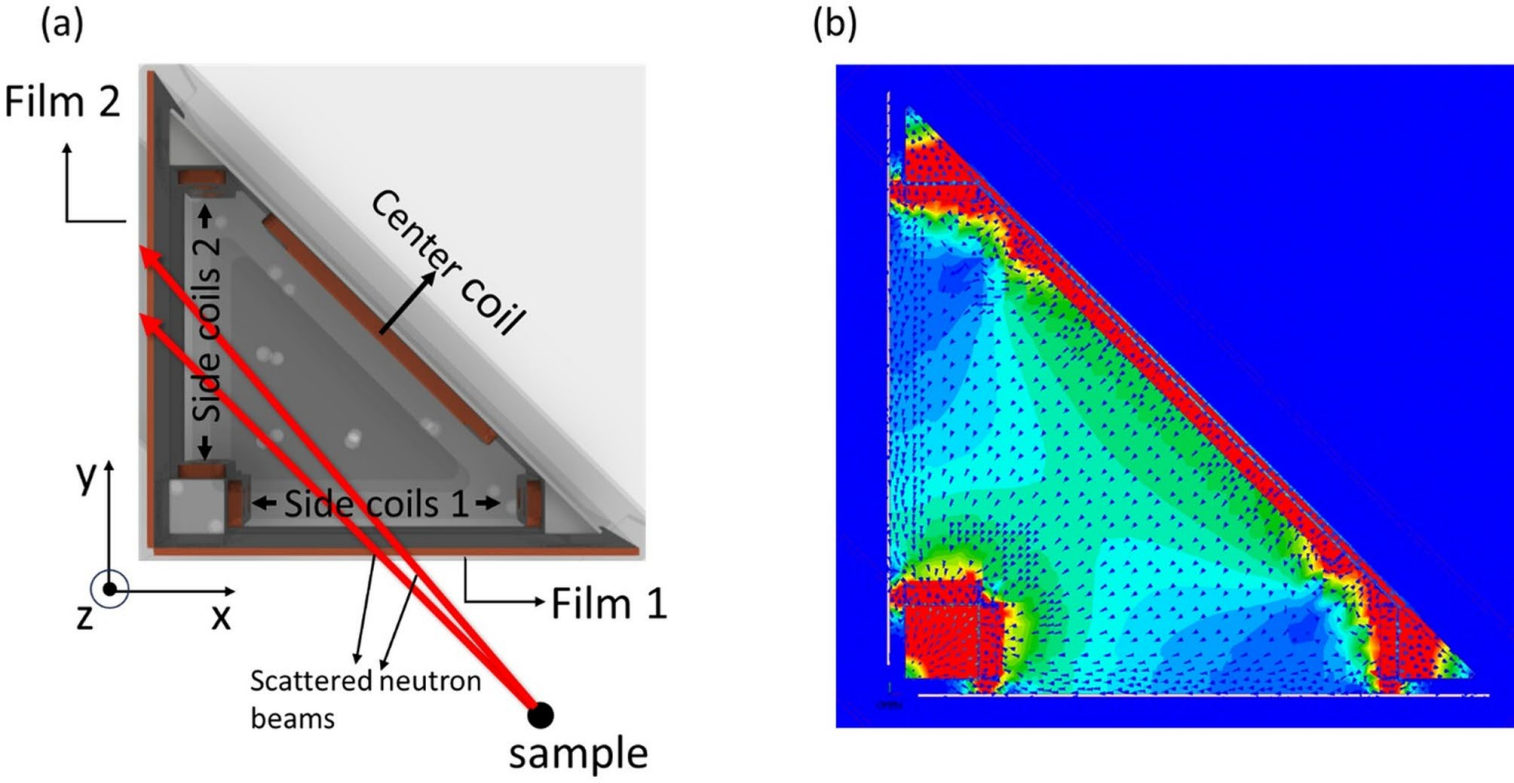
(**a**) The setup with two superconducting films forming a 90° angle. Three sets of coils are placed inside: two side coils and one center coil. (**b**) The simulated magnetic field with the three coils turned on.

In SNP, the neutron polarization ***P*** is treated as a classical vector, defined by any three orthogonal components. In principle, the scattered neutron polarization vector can be reconstructed if all three components are measured. Figure 8a shows a design that can make such measurements. It is composed of two superconducting films (100 mm x 90 mm) positioned at a 90 ° angle and three sets of independent coils mounted on copper frames, including two sets of side coils on the right-angled planes and one center coil on the opposite plane. The distance from the sample to the first superconducting film (Film 1) is at least 5 cm, limited by the diameter of the tail of our orange cryostat. Mu-metal shielding, which is not shown in the figure, is placed on the top and bottom of the frame as well as behind the center coil. The nutator field outside the second superconducting film (Film 2) can be oriented along *y* and *z* directions as defined in the figure. With all three sets of coils turned off, the y and z components (*P*_*y*_ and *P*_*z*_) of the scattered polarization can then be measured. To measure the *x* component, the three sets of coils are turned on to generate a magnetic field in between the two superconducting films (Figure 8b) so the x component (*P*_*x*_) can adiabatically rotate from film 1 to film 2. *P*_*x*_ can then be measured with the nutator still oriented along the y direction. The *P*_*x*_, *P*_*y*_ and *P*_*z*_ measurements assume the adiabatic condition is satisfied from the nutator to the analyzer.

**Fig. 9:**
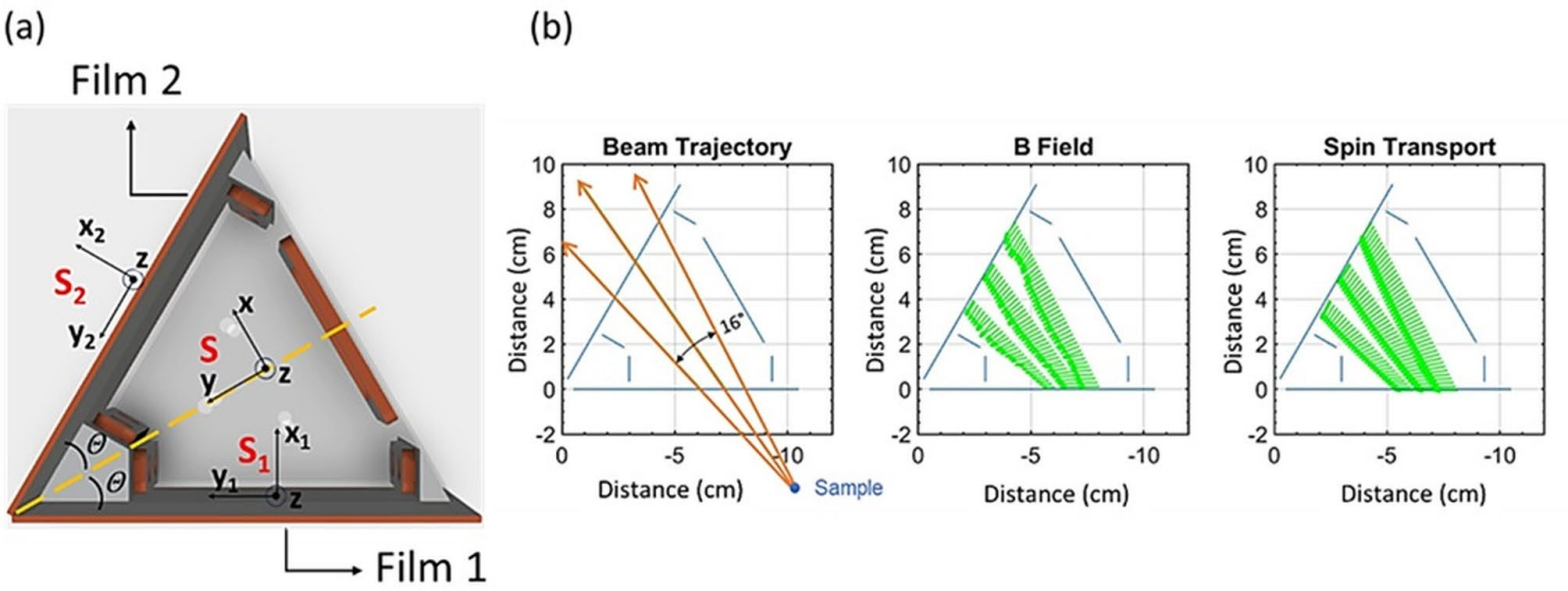
(**a**) The two superconducting films form a 60° angle to increase the angle coverage for the scattered beam, corresponding to $\theta$ = 30° (**b**) The range of neutron trajectories with satisfactory adiabatic conditions are shown when the three sets of coils are turned on. The corresponding magnetic fields and calculated spin transports for 15 meV neutrons are also presented.

The described 90° configuration of the two superconducting films presents a direct approach to measure the three orthogonal components of the scattered neutron polarization. However, this setup has a notable limitation: it offers a very narrow angular coverage of less than 5° while maintaining favorable adiabatic conditions for the scattered beam with an energy of 15 meV. One strategy to increase the angular coverage is to position the two films at a narrower angle. Figure 9a shows a setup with the two films forming a 60° angle while keeping the dimension of the films still the same (100 mm x 90 mm). In this setup, polarization components are derived from coordinate transformation. We define three coordinate systems – S, S_1_ and S_2_ – all sharing the same *z* direction. S_1_ and S_2_ are constructed such that *x*_*1*_ and *x*_*2*_ are normal to the surfaces of Film 1 and Film 2, respectively, with *y*_*1*_ and *y*_*2*_ parallel to these surfaces. For system S, its *x*-axis is perpendicular to an imaginary plane that bisects the angle between the films, as indicated by the yellow dashed lines in Figure 9a, while the y-axis aligns with these dashed lines. Again, with all three sets of coils turned off, the polarization components *P*_*z*_ and *P*_*y2*_ can be measured by aligning the nutator field along the z and y_2_ directions respectively. With the three coils turned on, assuming good adiabatic conditions from Film 1 to Film 2, *P*_*y1*_ can then be measured with the nutator field along the y_2_ direction. Since the polarization is a vector, *P*_*y1*_ and *P*_*y2*_ can be rewritten with *P*_*x*_ and *P*_*y*_ of the S system using a simple coordinate transformation:

1 $${P}_{y1}={P}_{x}\text{sin}\theta +{P}_{y}\text{cos}\theta$$

2 $${P}_{y2}={-P}_{x}\text{sin}\theta +{P}_{y}\text{cos}\theta ,$$

where $\theta$ equals to half of the angle between the two films. In the case in Figure 9, $\theta$ = 30°. From Eqs. (1) and (2) it is easy to derive:

3 $${P}_{x}=\frac{{P}_{y1}-{P}_{y2}}{2\text{sin}\theta }$$

4 $${P}_{y}=\frac{{P}_{y1}+{P}_{y2}}{2\text{cos}\theta }$$

With *P*_*x*_, *P*_*y*_ and previously measured *P*_*z*_, the scattered polarization vector ***P*** can then be uniquely determined.

With the 60° configuration, the angular coverage increases to about 16° for 15 meV neutrons. Figure 9b shows the range of the neutron trajectories, fields lines and corresponding spin transports. The range can be further extended with larger films and a smaller angle between the films. However, the wide angle coverage also requires a redesign of the downstream nutator and the zero-field chamber. We are working on a prototype based on this method, and the details will be presented in a separate work.

## Conclusion

SNP stands as a unique and powerful technique within the realm of neutron polarimetry, offering valuable complementary capabilities to conventional neutron scattering methods. The introduction of the newly developed SNP device, PHiTPAD, at ORNL, utilizing high-T_c_ superconducting YBCO films, a mu-metal zero-field chamber and a versatile orange cryostat, offers researchers at HFIR an additional tool to study magnetic structures and properties.

Furthermore, we have proposed a new method to facilitate SNP on the time-of-flight instrument HYSPEC at SNS. This approach permits the simultaneous measurement of scattered neutron polarizations across different scattering angles, whereas all the current SNP devices can only measure one angle at a time. The successful implementation of SNP on pulsed neutron sources holds the potential to broaden research horizons and foster new discoveries in the field.

## Data Availability

All relevant data are available from the corresponding author upon reasonable request.
